# CTLA4-Ig Effectively Controls Clinical Deterioration and Immune Condition in a Murine Model of Foxp3 Deficiency

**DOI:** 10.1007/s10875-023-01462-2

**Published:** 2023-05-08

**Authors:** Margaux Gerbaux, Evelyne Roos, Mathijs Willemsen, Frederik Staels, Julika Neumann, Leoni Bücken, Jeason Haughton, Lidia Yshii, James Dooley, Susan Schlenner, Stephanie Humblet-Baron, Adrian Liston

**Affiliations:** 1 KU Leuven, Department of Microbiology, Immunology and Transplantation, 3000 Leuven, Belgium; 2 Department of Medicine, Université Libre de Bruxelles, 1050 Brussels, Belgium; 3 VIB Center for Brain and Disease Research, 3000 Louvain, Belgium; 4 Immunology Programme, The Babraham Institute, Babraham Research Campus, Cambridge, CB22 3AT UK

**Keywords:** Inborn error of immunity, FOXP3 deficiency, Mouse model, Immunosuppressive treatment, IPEX

## Abstract

**Purpose:**

*FOXP3* deficiency results in severe multisystem autoimmunity in both mice and humans, driven by the absence of functional regulatory T cells. Patients typically present with early and severe autoimmune polyendocrinopathy, dermatitis, and severe inflammation of the gut, leading to villous atrophy and ultimately malabsorption, wasting, and failure to thrive. In the absence of successful treatment, FOXP3-deficient patients usually die within the first 2 years of life. Hematopoietic stem cell transplantation provides a curative option but first requires adequate control over the inflammatory condition. Due to the rarity of the condition, no clinical trials have been conducted, with widely unstandardized therapeutic approaches. We sought to compare the efficacy of lead therapeutic candidates rapamycin, anti-CD4 antibody, and CTLA4-Ig in controlling the physiological and immunological manifestations of Foxp3 deficiency in mice.

**Method:**

We generated Foxp3-deficient mice and an appropriate clinical scoring system to enable direct comparison of lead therapeutic candidates rapamycin, nondepleting anti-CD4 antibody, and CTLA4-Ig.

**Results:**

We found distinct immunosuppressive profiles induced by each treatment, leading to unique protective combinations over distinct clinical manifestations. CTLA4-Ig provided superior breadth of protective outcomes, including highly efficient protection during the transplantation process.

**Conclusion:**

These results highlight the mechanistic diversity of pathogenic pathways initiated by regulatory T cell loss and suggest CTLA4-Ig as a potentially superior therapeutic option for FOXP3-deficient patients.

**Supplementary Information:**

The online version contains supplementary material available at 10.1007/s10875-023-01462-2.

## Introduction

Immune dysregulation polyendocrinopathy and enteropathy X-linked (IPEX) syndrome is a rare autoimmune disorder caused by monogenic mutation of *FOXP3*, crucial for the development and function of regulatory T cells (Treg) [1–4]. Patients present with early and severe multiorgan autoimmunity, including the typical triad of autoimmune polyendocrinopathy with autoimmune diabetes, dermatitis, and severe refractory watery diarrhea caused by inflammation of the gut, leading to villous atrophy and ultimately malabsorption, wasting and failure to thrive. The presentation is highly variable, including other common manifestations such as autoimmune cytopenia, hepatitis, nephritis, hypo- or hyperthyroidism, arthritis, interstitial lung disease, and less frequent lymphadenopathy, splenomegaly, or food allergy [1–7]. The risk of infection is increased, as a consequence of the alteration of the normal digestive and cutaneous barrier, undernutrition, and immunosuppressive treatment [1–4]. Biologically, IPEX is characterized by elevated IgE, eosinophilia, and autoantibodies, in addition to the causative absence of Tregs. Depending on the nature of the *FOXP3* mutation, FOXP3^+^ Tregs can be absent, reduced, or in the normal range but with impaired suppressive function [1, 2, 4–6, 8, 9].

In the absence of appropriate diagnosis and treatment, IPEX patients usually die within the first 2 years of life [1, 2, 8, 10]. The only curative therapeutic option is hematopoietic stem cell transplantation (HSCT) [1, 2, 5, 6, 11]; however, both symptomatic treatment and immunosuppressive treatment are typically required prior to HSCT. Symptomatic treatment includes hormone replacement therapy (insulin, thyroid hormone), nutritional support such as parenteral nutrition in case of severe enteropathy, and intravenous immunoglobulin replacement therapy if required. Immunosuppressive treatment is required to prevent further spread of autoimmune damage and tissue degeneration. The therapeutic approach for immunosuppression remains unstandardized, with the most common treatments being the calcineurin inhibitors cyclosporine A and tacrolimus and the mTOR inhibitor rapamycin, combined or as monotherapy, with or without steroid treatment [1–3, 6, 10]. Biologics have recently been offered as a potential new therapeutic option, mostly supported by their immune rationale, mouse model, or anecdotal human reports [3, 5, 12–17]. The proposed treatments include anti-CD4 antibodies and soluble CTLA4-Ig fusion protein abatacept. While originally proposed to work through increasing Treg activity [13, 14, 18–22], anti-CD4 antibodies also induced tolerance in Foxp3/Treg-deficient mice, with a benefit observed in terms of dermatitis and multisystemic autoimmune manifestations, suggesting a Treg-independent mechanism based on anergy or deletion of autoreactive T cells [12]. CTLA4-Ig functions in a different manner, competitively binding CD80/CD86 on antigen presenting cells, leading to T cell anergy [3, 15, 16]. It has already been successfully used to control inflammation in specific monogenic autoimmune diseases such as CTLA-4 or LRBA deficiency [3, 23, 24] and based on mode of activity is a valid candidate to control IPEX disease.

Due to the rarity of IPEX disease, no standardized clinical trials have been conducted so far and the therapeutic approach remains widely unstandardized. Mouse models can therefore serve to prioritize treatment selection. The mouse equivalent of IPEX patients is Foxp3-deficient mice, caused by mutations or deletions in the *Foxp3* gene. As with IPEX patients, Foxp3 deficiency causes an absence of functional Foxp3^+^ Tregs, leading to multiorgan lymphoid and myeloid infiltration with systemic autoimmune manifestations comparable to those of IPEX in human [1, 4, 25]. Here, we sought to compare the efficacy of rapamycin, nondepleting anti-CD4 antibody, and CTLA4-Ig in controlling the physiological and immunological manifestations of Foxp3 deficiency in mice. We found selective suppression of distinct aspects of pathology, demonstrating that Foxp3 deficiency is driven by multiple distinct pathophysiological processes. In addition to highlighting the mechanistic divergence occurring in the Foxp3-deficient context, these studies identify CTLA4-Ig as a potential therapeutic in IPEX, showing superior breadth of control over pathology.

## Methods

### Mice

*Foxp3*^*KO*^ mice were generated on the E14 ES background. A transcriptional stopper cassette, consisting of two SV40 polyadenylation sites flanked by LoxP sites, was introduced by conventional gene targeting in E14 ES cells between exons 4 and 5 of the mouse *Foxp3* gene. The gene targeting vector contained an FRT-flanked Neomycin resistance gene which was excised by crossing to Flpase germline deleter mice (Gt(ROSA)26Sortm1(FLP1)Dym/J (Stock No. 003946)) (Supplementary Figure S1A, B). The Foxp3-Stop allele was backcrossed to C57Bl/6 for more than 10 generations and maintained on a Rag2-deficient background. *Foxp3*^*KO/KO*^.*Rag2*^*KO/KO*^ female mice were bred to wild-type male mice to generate *Foxp3*^*KO*^ male mice. This breeding strategy enables a *Foxp3*^*KO*^ colony to be maintained without the generation of surplus *Foxp3*^*KO*^ mice, as *Foxp3*^*KO*^ stock is only produced when desired, by outbreeding. While providing ethical advantages, this breeding strategy results in *Foxp3*^*KO*^ mice that are also *Rag*^*KO/WT*^ heterozygous. While a potential confounder, no cellular or inflammatory phenotype has been observed for Rag heterozygosity, and both untreated and treated mice had the same genotype. *Foxp3*^*KO*^ mice were treated with rapamycin (Sigma), CTLA4-Ig (Abatacept, Orencia® (BMS)), or nondepleting anti-CD4 antibody (YTS177.9, BioXcell) starting at day 7 of age. Rapamycin was given at a dose of 4 mg/kg every 2 days, CTLA4-Ig was given at a dose of 25 mg/kg every 4 days, and anti-CD4 antibody was given at a dose of 10 mg/kg every 2 days. Clinical condition was scored based on the aggregate of spleen enlargement, runting, skin condition, eye condition, tail condition, ear condition, and gait, as in Table S1. Mice evaluated as severely ill, as per the University of Leuven ethics guidelines, were euthanized. For bone marrow transplantation, mice were irradiated with 4 Gy total body irradiation, followed by transplantation of 25 × 10^6^ bone marrow cells from wild-type mice. Mice were maintained in conventional facilities of the University of Leuven. All experiments were approved by the University of Leuven Ethics Committee.

### Flow Cytometry

Single-cell suspension was prepared from mouse thymus, bone marrow, spleen, and pooled peripheral lymph nodes (cervical, inguinal, axillary, and brachial). For intracellular cytokine staining, cells were plated at 5 × 10^5^ cells/well in 96-well tissue cultures plates in complete RPMI containing phorbol 12-myristate 13-acetate (50 ng/mL; Sigma-Aldrish), ionomycin (250 ng/mL; Sigma-Aldrish), and monensin (1/1500; BD Biosciences, San Jose, Calif) for 4 h at 37 °C. Cells were fixed with BD Cytofix (BD Biosciences) or fixed and permeabilized with the eBioscience FoxP3 staining kit (eBioscience/Affymetrix, San Diego, Calif). Anti-murine antibodies included anti-CD4 (RM4-5), anti-CD8a (53–6.7), anti-CD25 (PC61.5), anti-CTLA4 (UC10-4B9), anti-CD44 (IM7), anti-CD62 ligand (MEL-14), anti-CD19 (eBio1D3), anti-CD3 (145-2c11), anti-T-bet (4B10), anti-IFNγ (XMG1.2), and anti-IL-4 (BVD6-24G2) from eBioscience. Representative gating profiles are shown in Supplementary Fig. 2.

### Histology

Mice were sacrificed at day 26, and mouse tissues were preserved in 10% formalin and processed into paraffin-embedded tissue blocks by Histology Consultation Services (Everson, Wash). Each block had thin (approximately 4-mm) sections cut on a microtome, mounted on glass slides, and stained with hematoxylin and eosin. Pathological diagnosis was performed by Biogenetics Research Laboratories, Greenbank, Washington. Lesions were graded from absent to marked (Table S2).

### Serum Analysis

IgE titers in individual serum sample were determined by using a mouse IgE ELISA Ready-SET-Go! Kit (eBioscience), according to the manufacturer’s protocol. The concentration of Rapamycin in serum was assessed in the clinical laboratory of UZ Leuven by liquid chromatography with tandem mass spectrometry.

### Statistical Analysis

All statistical analyses were carried out with GraphPad Prism (GraphPad Software). Ordinary one-way ANOVA was used to compare multiple independent groups, followed by Tukey’s multiple comparison test. For the analysis of less than 6 data points, normality was assessed on residuals. Ordinary two-way ANOVA with a mixed effects model was used to compare multiple groups with repeated measures over time. For survival curves, a log-rank (Mantel-Cox) test was applied to compare all groups, followed by multiple comparison between each groups with the method of Holm Sidak (setting alpha at 0.05), accounting for multiple comparison.

## Results

### Divergent Responses to Different Treatment Regimes in Foxp3-Deficient Mice

**Fig. 1:**
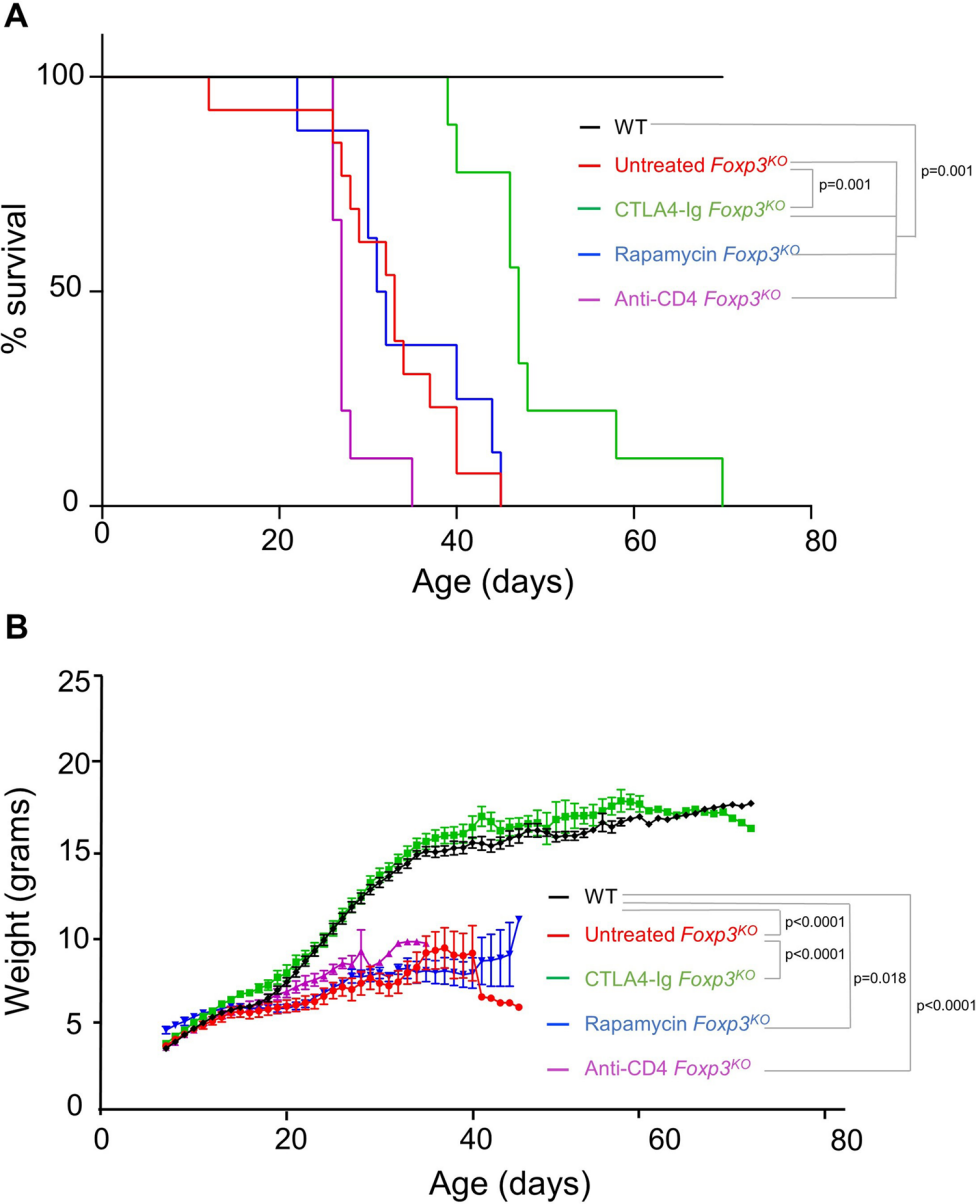
CTLA4-Ig treatment improves survival and abolish wasting in *Foxp3*^*KO*^ mice. *Foxp3*^*KO*^ mice and wild-type littermate controls (WT) were followed up for a longitudinal study without therapeutic intervention (WT, *n* = 13; *Foxp3*.^*KO*^, *n* = 13) or following treatment with rapamycin (*n* = 8), CTLA4-Ig (*n* = 9), or anti-CD4 antibody (*n* = 9). **A** Mice were evaluated daily and were culled upon reaching threshold severity; curve shows subthreshold survival. **B** Weight curve (mean ± SEM)

To perform a comparative test of potential IPEX therapeutics, we generated a new *Foxp3*^*KO*^ strain, using a Cre-reversible insertion of STOP sequences between exons 4 and 5 of the *Foxp3* locus. Proof of Foxp3 deficiency was assessed by absent Foxp3 expression in *Foxp3*^*KO*^ mice compared to positive expression in wild-type controls (Supplementary Fig. 1C). As with other *Foxp3*^*KO*^ or mutant strains, the *Foxp3*^*KO*^ strain developed analogous clinical manifestations to IPEX patients, scaly skin (especially upon the ears, eyes, and tail), runting, and premature death at an average of 33 days (Fig. 1). To determine the global impact of our three treatment regimes, we treated *Foxp3*^*KO*^ mice with rapamycin, CTLA4-Ig, or nondepleting anti-CD4 antibody, from 7 days of age (Supplementary Figure S3A). Rapamycin treatment was confirmed by assessing the concentration of rapamycin in blood 24 and 48 h after the last injection (Supplementary Figure S3B). No increase in life expectancy was observed in the rapamycin- or anti-CD4 antibody-treated mice (at 31.5 and 27 days, respectively), while CTLA4-Ig substantially increased the healthy lifespan out to 47 days (Fig. 1A). These results were mirrored by weight-change measurements, where rapamycin- and anti-CD4 antibody-treatment provided no benefit to the severe runting experienced by *Foxp3*^*KO*^ mice (Fig. 1B). By contrast, CTLA4-Ig-treated *Foxp3*^*KO*^ mice demonstrated normal weight gain, even when they reached euthanasia thresholds for other symptoms (Fig. 1B).

**Fig. 2:**
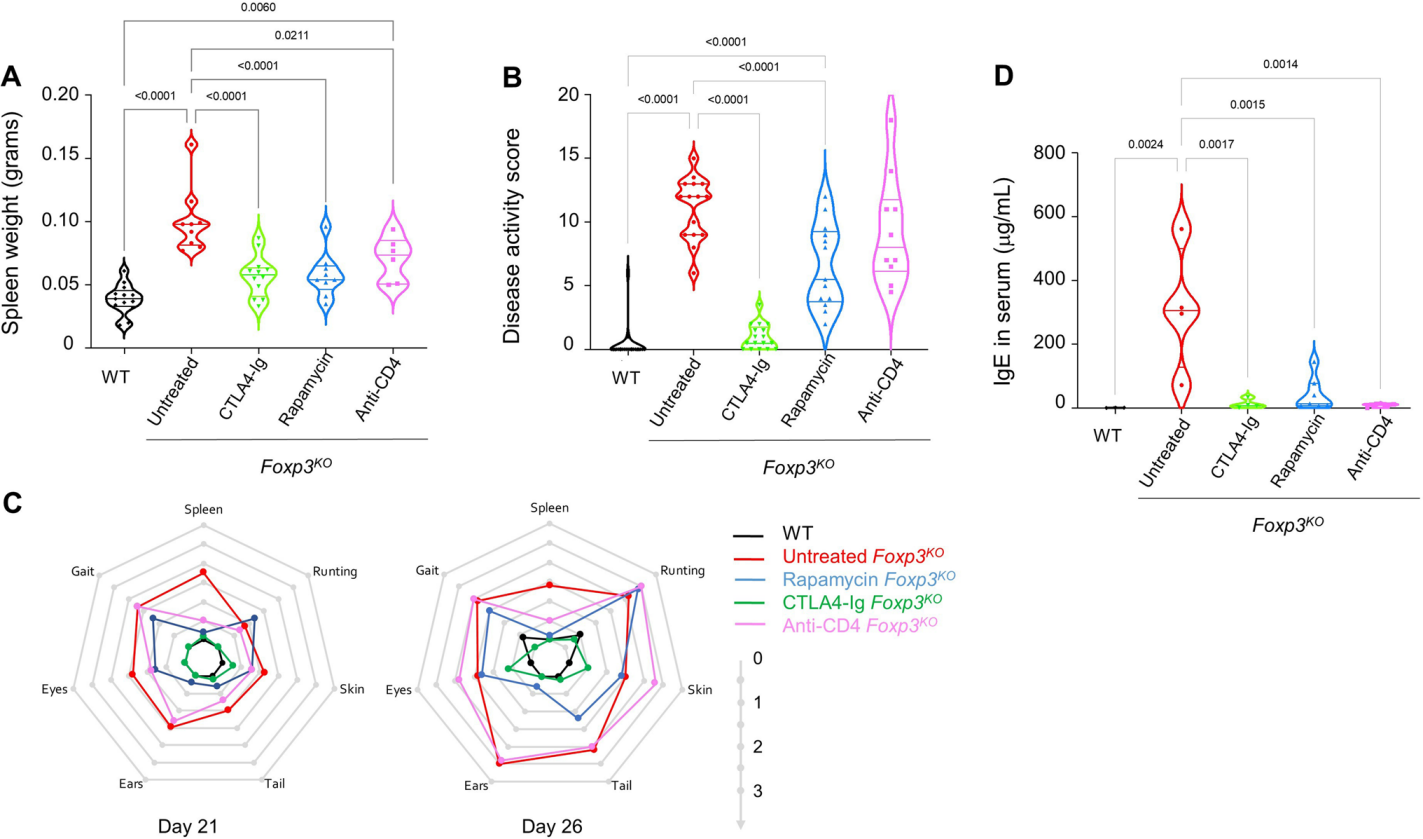
Divergent clinical benefits of selected therapeutics identify distinct pathways of pathology in *Foxp3*^*KO*^ mice. *Foxp3*^*KO*^ mice, untreated or treated with rapamycin, CTLA4-Ig, or anti-CD4 antibody, as well as untreated wild-type littermate controls (WT), were assessed at day 21 or 26. **A** Spleen weight, pooled from day 21 and 26 (WT, *n* = 13; *Foxp3*^*KO*^ untreated, *n* = 9; *Foxp3*^*KO*^ rapamycin, *n* = 9; *Foxp3*^*KO*^ CTLA4-Ig, *n* = 12; or *Foxp3*^*KO*^ anti-CD4 antibody, *n* = 6). **B** Aggregate disease activity score for days 21 (WT, *n* = 13; *Foxp3*^*KO*^ untreated, *n* = 8; *Foxp3*^*KO*^ rapamycin, *n* = 6; *Foxp3*^*KO*^ CTLA4-Ig, *n* = 9; or *Foxp3*^*KO*^ anti-CD4 antibody, *n* = 6) and 26 (WT, *n* = 8; *Foxp3*^*KO*^ untreated, *n* = 7; *Foxp3*^*KO*^ rapamycin, *n* = 7; *Foxp3*^*KO*^ CTLA4-Ig, *n* = 8; or *Foxp3*^*KO*^ anti-CD4 antibody, *n* = 4). Violin plot with individual values for **A** and **B**. **C** Radial plots for the average disease score across the seven composite disease activity measures, for day 21 (left) and day 26 (right). **D** Serum IgE levels (WT, *n* = 3; *Foxp3*^*KO*^ untreated, *n* = 4; *Foxp3*^*KO*^ rapamycin, *n* = 7; *Foxp3*^*KO*^ CTLA4-Ig, *n* = 4; or *Foxp3*.^*KO*^ anti-CD4 antibody, *n* = 4)

In the aging cohort and postmortem analysis, we observed partial restoration of clinical phenotypes in treated mice, not reflected in the healthy survival and weight-change measurements. We therefore developed a clinical scoring system for Foxp3 deficiency, using a 0–3 scoring system covering splenomegaly (Fig. 2A), weight-change (Fig. 1B), behavior changes (in particular, hunching), and separate measures of dermal thickening and redness on the skin, tail, ears and around the eyes (Supplementary Table 1). We then aged additional cohorts of *Foxp3*^*KO*^ mice and compared treated and untreated mice at day 21 and day 26 of age. Aggregate clinical scores demonstrated a near-complete correction of pathology in CTLA4-Ig-treated mice, while rapamycin-treated mice exhibited a ~ 50% protection and anti-CD4 antibody-treated mice only a nonsignificant trend in protection (Fig. 2B). These aggregate scores, however, obscure the substantial heterogeneity in response per organ system. Displaying the individual clinical values demonstrated that rapamycin provided near-complete protection against splenomegaly and dermal pathology around the ears and partial protection in dermal pathology of the other sites, with little effect on gait and no effect on runting (Fig. 2C). Anti-CD4 antibody treatment, while having little effect on most measures, was highly effective at preventing splenomegaly (Fig. 2C). Finally, CTLA4-Ig treatment was effective across all the scored tissues (Fig. 2C). All treatments controlled serum IgE levels (Fig. 2D).

**Fig. 3:**
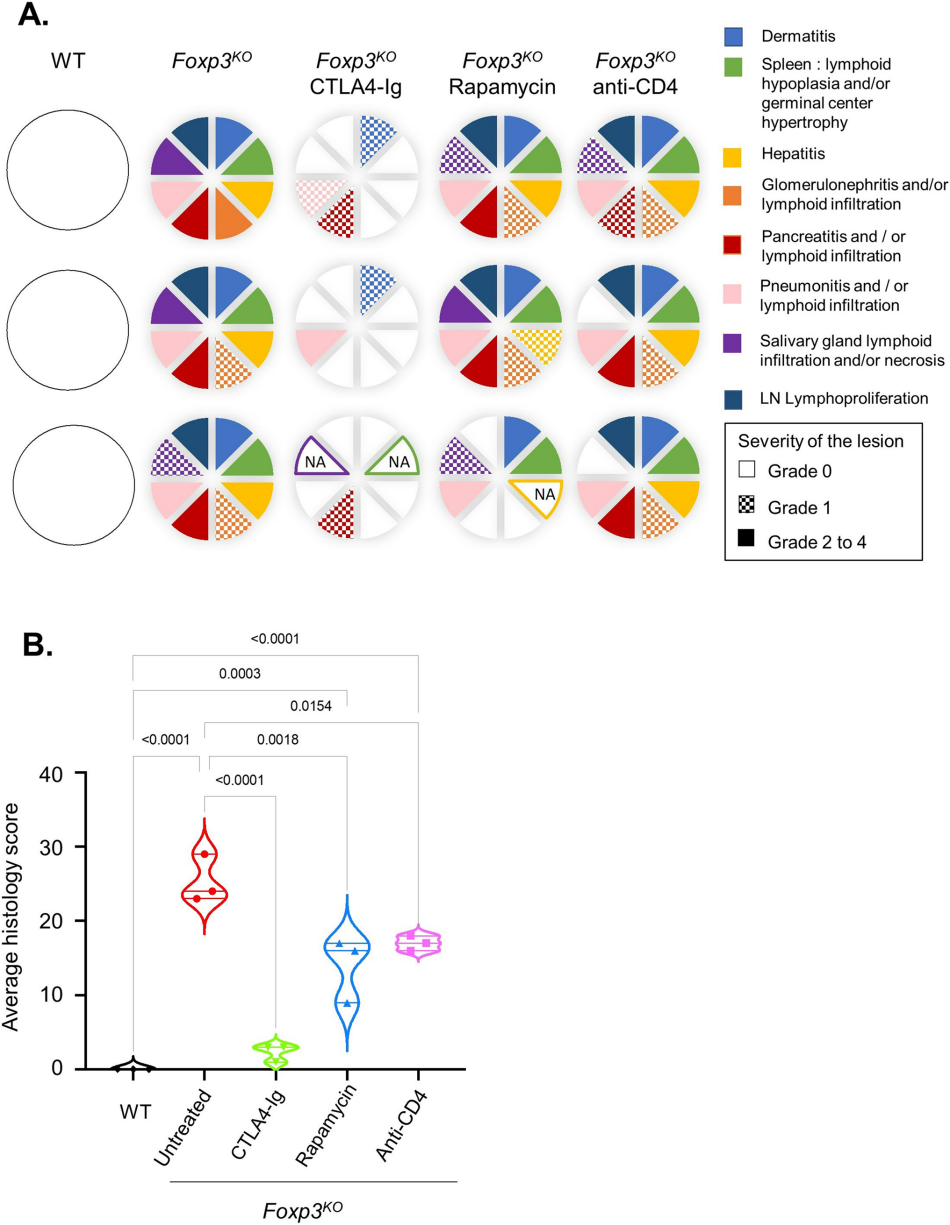
Widespread protection against organ damage in CTLA4-Ig-treated Foxp3-deficient mice. *Foxp3*^*KO*^ mice, untreated or treated with rapamycin, CTLA4-Ig, or anti-CD4 antibody, as well as untreated wild-type littermate controls (WT), were assessed at day 26 for histopathology (*n* = 3/group). Scoring was performed on the lungs, spleen, liver, kidneys, pancreas, salivary glands, skin, lymph nodes, jejunum, and ilium to monitor pathological observations. **A** Each circle represents histopathological examination of an individual mouse scored from absent (no color), to minimal (grade 1: grid), to mild or above (grades 2 to 4: solid). No abnormality was observed in ilium and jejunum (not represented). NA: not available. **B** Aggregate histopathological scores (*n* = 3/group). Violin plot with individual values

We next performed a histological survey covering ten organs in treated and untreated mice at 26 days of age. *Foxp3*^*KO*^ mice, as in other knockout and mutant strains, developed a multiorgan lymphoid and myeloid infiltration (Fig. 3A). CTLA4-Ig-treated mice, while being protected from the clinical measures used, still developed mild histological dermatitis, pneumonitis and pancreatitis, in some mice (Fig. 3A). Rapamycin- and anti-CD4 antibody-treated mice did not show a consistent organ-specific pattern of protection; however, a net decrease in histopathology was observed (Fig. 3B). Together, these results indicate a strong, but incomplete, pathological protection via CTLA4-Ig, and weaker effects for rapamycin and anti-CD4 antibodies.

**Fig. 4:**
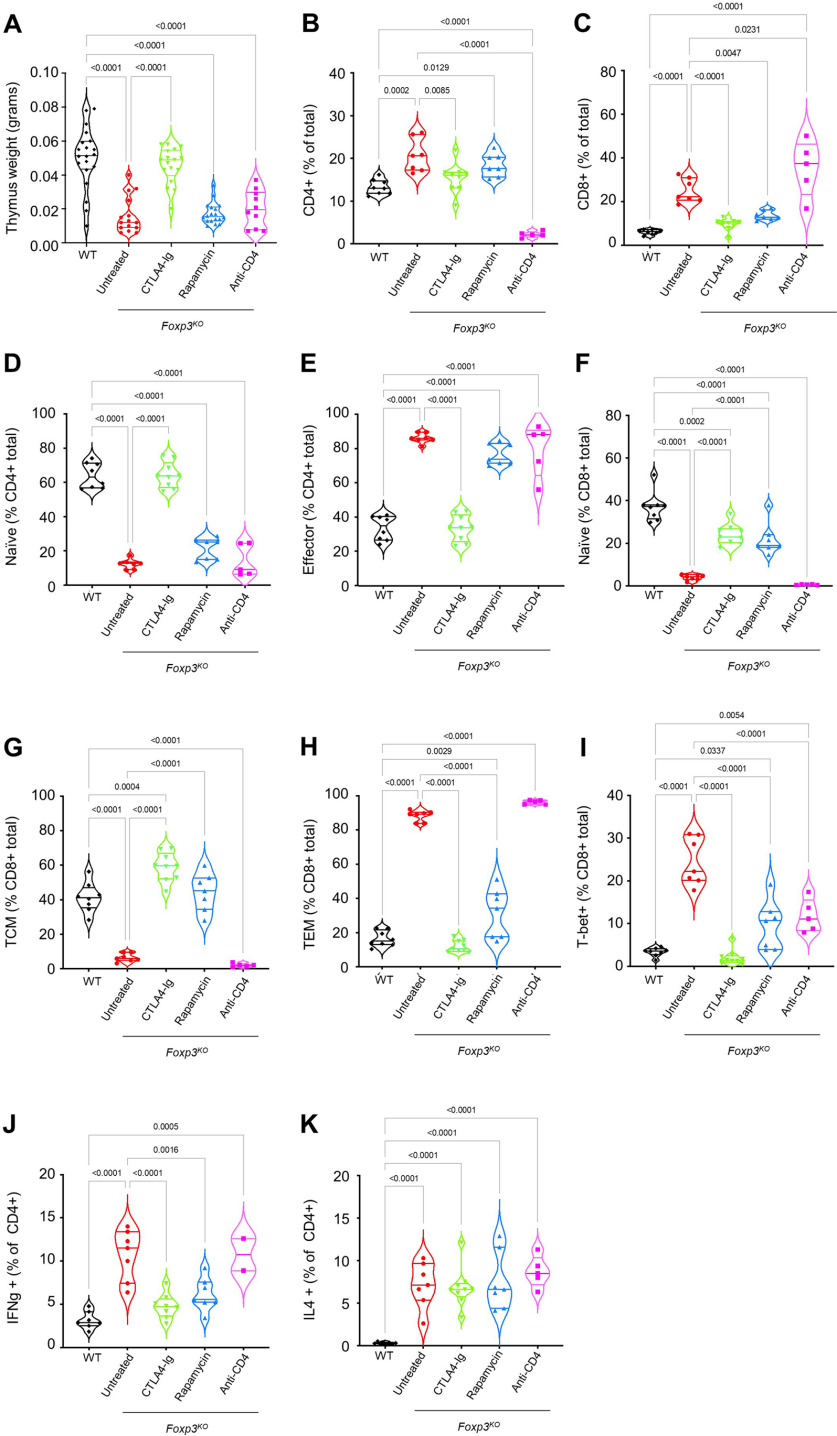
Diverging immunological outcomes of different treatments in Foxp3-deficient mice. *Foxp3*^*KO*^ mice, untreated or treated with rapamycin, CTLA4-Ig, or anti-CD4 antibody, as well as untreated wild-type littermate controls (WT), were assessed by flow cytometry on day 26 (WT, *n* = 8; *Foxp3*^*KO*^ untreated, *n* = 8; *Foxp3*^*KO*^ rapamycin, *n* = 6; *Foxp3*^*KO*^ CTLA4-Ig, *n* = 9; or *Foxp3*^*KO*^ anti-CD4 antibody, *n* = 5). **A** Thymus weight. **B** The percentage of CD4^+^ T cells or **C** CD8^+^ T cells from total splenocytes. **D** Percentage of naïve (CD44^−^) and **E** effector (CD44^+^) T cells from CD4^+^ splenocytes. **F** Percentage of naïve (CD44^−^CD62L^+^), **G** central memory (TCM) (CD44 + CD62L^+^), **H** TEM (CD44 + CD62L^−^), and **I** Tbet^+^ cells from CD8^+^ splenocytes. **J** Percentage of CD4^+^ T cells in the spleen expressing IFNγ or **K** IL4. Violin plot with individual values

In order to determine the mechanism of action of the immunosuppressive effects, we profiled the immune system changes occurring during Foxp3 deficiency and treatment. *Foxp3*^*KO*^ mice presented with a thymic cellularity collapse, indicative of cytokine storm (Fig. 4A) and profound activation of T cells in the spleen (Fig. 4B–J, Supplementary Fig. 4) and lymph nodes (Supplementary Figure S5). Expansion of both CD4 (Fig. 4B) and CD8 (Fig. 4C) populations was observed, with the naïve and memory populations restricted at the expense of effector cells (Fig. 4D–H). Among the expanded effector cells were large increases in the Tbet^+^ CD8 T cell population (Fig. 4I) and both Th1 and Th2 cells in the CD4 lineage (Fig. 4J, K). Rapamycin corrected most CD8 T cell phenotypes, preserving naïve and central memory cells (Fig. 4F, G) and preventing the excessive expansion of effector memory and Tbet^+^ cells (Fig. 4H, I). The effect of anti-CD4 antibody treatment, by contrast, was largely limited to restricting the total number of CD4 T cells (Fig. 4B). CTLA4-Ig treatment prevented the development of almost all T cell phenotypes in *Foxp3*^*KO*^ mice, with the exception of elevated Th2 numbers. CTLA4-Ig treatment also demonstrated a substantial but not statistically significant effect, on CD80 expression on inflammatory myeloid cells (Supplementary Fig. 6A), consistent with known biological effects, and mitigation of the CTLA4 upregulation by conventional T cells normally observed in Foxp3KO mice (Supplementary Fig. 6B), an effect potentially due to the general control over inflammation. These results suggest a mechanistic basis for the disparate clinical effects observed, with the distinct immunological impacts a potential explanation for the distinct clinical effects.

### CTLA4-Ig Improves Survival and Conditioning Following HSCT

**Fig. 5:**
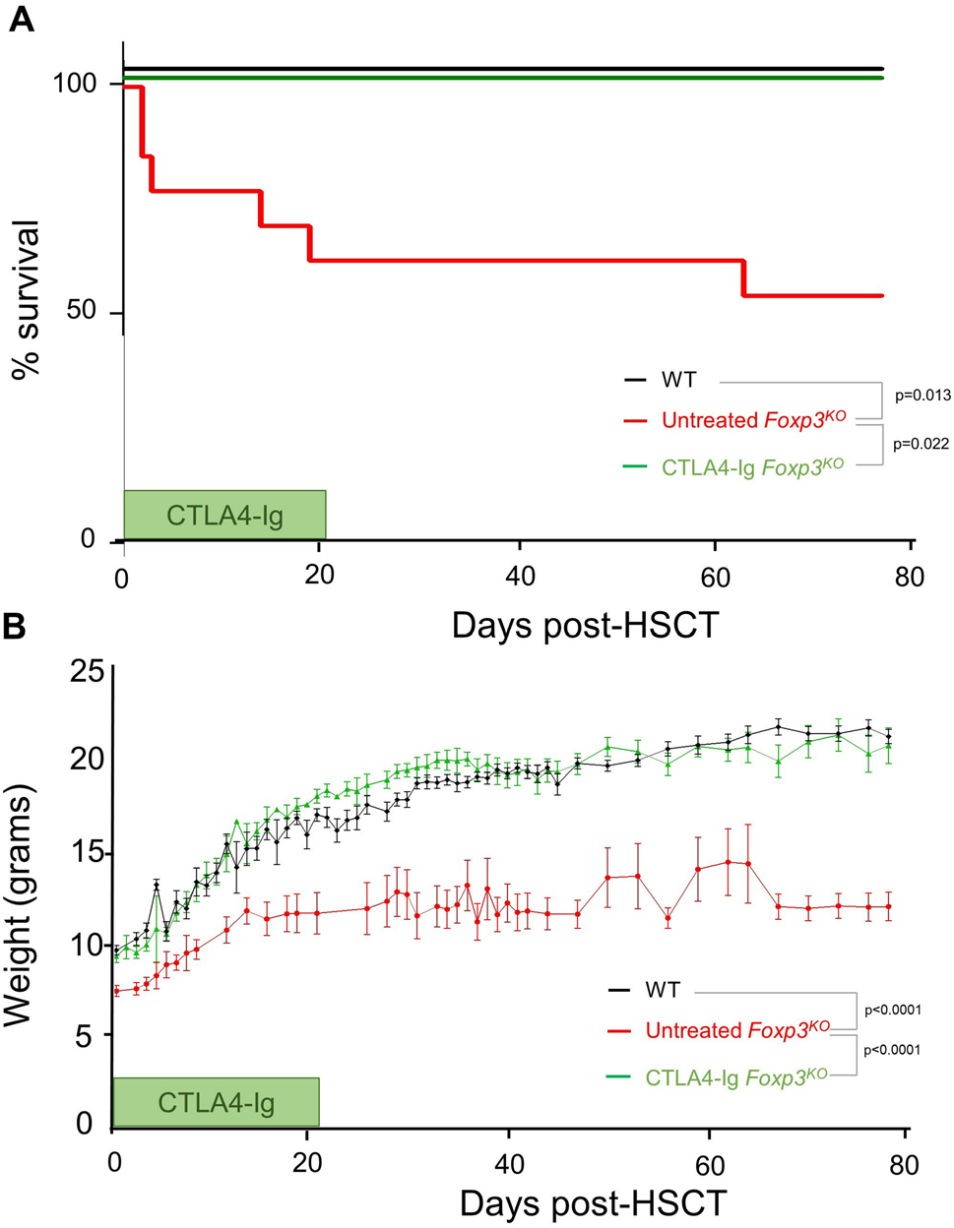
CTLA4-Ig treatment of Foxp3^KO^ mice improves survival and clinical condition during HSCT. Wild-type littermate controls (*n* = 14) and *Foxp3*.^*KO*^ mice, untreated or treated with CTLA4-Ig (*n* = 12.11), were given HSCT at 25 days of life. **A** Mice were evaluated daily and were euthanized upon reaching threshold severity; curve shows subthreshold survival. **B** Weight curve (mean ± SEM)

Inflammation is a negative prognostic indicator for engraftment and survival after HSCT, providing a major barrier for the only curative treatment available for IPEX. The profound benefits observed here in *Foxp3*^*KO*^ mice treated with CTLA4-Ig suggested a potential use in the clinical context of preparing IPEX patients for HSCT. We therefore performed a CTLA4-Ig preconditioning study, whereby *Foxp3*^*KO*^ mice were either left untreated prior to transplantation, or were treated with CTLA4-Ig from one week of age. Both groups were given a HSCT at 25 days of age, with the CTLA4-Ig group continuing on treatment until 3 weeks posttransplant. 42% of the untreated *Foxp3*^*KO*^ mice reached clinical endpoint, with many requiring euthanasia in the week following HSCT (Fig. 5A), while the surviving mice remained runted even after successful engraftment (Fig. 5B). By contrast, CTLA4-Ig treatment prior to transplantation ensured mice was in a sufficient condition to prevent adverse events during conditioning, allowed for successful engraftment, and resulted in long-term improved survival and healthy weight gain (Fig. 5A, B). These results suggest that the clinical gains of CTLA4-Ig treatment observed in *Foxp3*^*KO*^ mice protect against the deleterious effects of bone marrow transplantation. Treatment with CTLA4-Ig therefore synergizes with the potentially curative context of HSCT, providing a safe and efficacious combination for the treatment of Foxp3 deficiency.

## Discussion

The comparative analysis of three immunosuppressive agents in *Foxp3*^*KO*^ mice—rapamycin, nondepleting anti-CD4 antibody, and CTLA4-Ig—demonstrated qualitative rather than quantitative differences in pathology suppression. At a clinical level, rapamycin notably improved splenomegaly and partially skin pathology, in particular around the ears, while at the cellular level rapamycin inhibited excessive CD8 T cell responses. CD4 responses, by contrast, remained largely uninhibited, with the exception of IFNγ production (known to be dependent on the rapamycin-sensitive mTORC1 complex [26, 27]). The dominant view of rapamycin activity is that the effects are felt through inhibiting CD4 effector T cell responses while inducing Treg activity [28, 29]. By contrast, the effect of rapamycin on CD8 T cells is complex and can even act in an immunostimulatory manner on memory CD8 formation [30]. The predominant CD8 T cell correction observed here suggests that the immunosuppressive influence of rapamycin is shifted due to the lack of Tregs in the Foxp3-deficient system. Nondepleting anti-CD4 antibody treatment, by contrast, exhibited negligible effects on CD8 T cells, with the dominant immunological phenotype observed being a reduction in CD4 T cell numbers. While preventing splenomegaly and reducing histological inflammation, nondepleting anti-CD4 antibody treatment was unable to prevent runting, dermatitis, or premature lethality. The tolerogenic effect of nondepleting anti-CD4 antibodies has been long demonstrated and is thought to rely on promoting anergy and Treg induction [13, 14, 18–22]. The recent observation of treatment-induced tolerance induction in scurfy mice [12] suggests a function independent of Tregs, although the effect was only transient and partially effective in controlling inflammation. Notably, while assessment in *scurfy* mice was effective at controlling dermatitis and tissue inflammation, the effect on survival was not assessed in *scurfy* mice, but only in deletion and transfer systems [12]. Our current work recapitulates the anti-inflammatory effect of anti-CD4 antibody treatment on tissue inflammation, although not on dermatitis. The differential effects on survival: negligible on Foxp3-deficient mice (this study), subtle in adult *Foxp3*^*DTR*^ mice treated with diphtheria toxin [12], and substantial in an adoptive transfer model of scurfy [12], suggest that anti-CD4 antibody treatment is more effective in milder forms of Treg-deficiency. We note that while the anti-CD4 antibody used here is considered nondepleting, the prolonged treatment resulted in a strong reduction in CD4 T cells. Finally, CTLA4-Ig treatment provided the strongest benefits to *Foxp3*^*KO*^ mice. CTLA4-Ig was the only treatment to improve all clinical parameters measured and almost all immunological parameters, with the exception of Th2 numbers. Strikingly, CTLA4-Ig was the only treatment with any effect on weight loss, which was completely corrected in treated Foxp3-deficient mice. CTLA4-Ig may substitute in part for the lack of endogenous CTLA4 expression by Tregs. While the key function of endogenous Treg CTLA4 in trans-endocytosis of costimulation [31] is not replicated by CTLA4-Ig, the fusion protein competitively binds CD80/CD86, repressing CD28 costimulation in T cells [3, 15, 16]. In addition to its widespread usage in common autoimmune conditions, CTLA4-Ig has already been successfully used to control inflammation in primary immunodeficiencies such as CTLA4 or LRBA deficiency [3, 23, 24], with our results here suggesting it may additionally serve to control IPEX disease. It is notable here that the differences in treatment response were qualitative rather than quantitative; i.e., at the endophenotype level, each of rapamycin, anti-CD4 antibody, and CTLA4-Ig provided a near-digital effect, either reversing or sparing the measure. This demonstrates that the different aspects of Foxp3 deficiency pathology measured here can have exclusive mechanisms operating, indicative of multiple independent tolerogenic roles for Tregs.

HSCT provides a full curative therapeutic option for IPEX. There is, however, still a need for effective immunosuppression. For most patients, immunosuppression prior to transplantation is required to prevent further degeneration while a donor is identified [1, 5, 6, 11]. Alternatively, *FOXP3* mutations are occasionally found in older patients with milder disease, often associated with partial-loss-of-function mutations, in whom effective immunosuppression would provide a lower risk than HSCT. Finally, even among young patients with an available donor, immunological dysfunction and poor clinical condition prior to HSCT are associated with higher risk of complications and mortality [1, 5]. Clinical stability and control of inflammation with the use of appropriate immunosuppressive drugs prior to HSCT are thus crucial for successful engraftment and minimization of transplant-related complications [1, 5]. With low patient number precluding comprehensive clinical trials, selection of the appropriate immunosuppressive regime to complement HSCT is rather empirical but could depend, in part, on mouse models. Here, CTLA4-Ig treatment was the standout not only in the prevention of the broadest range of clinical and immunological parameters in *Foxp3*^*KO*^ mice but also in the superior outcome in engraftment and condition following HSCT. These results suggest that CTLA4-Ig treatment should be considered for IPEX patients who demonstrate poor outcome under treatment with standard immunosuppressive drugs such as rapamycin.

## Supplementary Information

Below is the link to the electronic supplementary material.

Supplementary file1 (PDF 1091 KB)

## Data Availability

The datasets generated during and/or analyzed during the current study are available from the corresponding author on reasonable request.
