# Supplementary material for: CTLA4-Ig Effectively Controls Clinical Deterioration and Immune Condition in a Murine Model of Foxp3 Deficiency
Source: J Clin Immunol. 2023 May 8;43(6):1393–402. doi: 10.1007/s10875-023-01462-2 (PMC10354160; doi:10.1007/s10875-023-01462-2)

Supplementary materials

CTLA4-Ig effectively controls clinical deterioration and immune condition in a murine model of Foxp3-deficiency

Margaux Gerbaux[1,2*], Evelyne Roos[1,3*], Mathijs Willemsen[1,3], Frederik Staels[1,3], Julika Neumann[1,3], Leoni Bücken[1], Jeason Haughton[1], Lidia Yshii, James Dooley[1,3,4], Susan Schlenner[1], Stephanie Humblet-Baron[1#], and Adrian Liston[1,3,4#]

[1] KU Leuven, Department of Microbiology, Immunology and Transplantation, Leuven 3000, Belgium.

[2] Université Libre de Bruxelles, Department of Medicine, 1050 Bruxelles, Belgium.

[3] VIB Center for Brain and Disease Research, Leuven 3000, Belgium.

[4] Immunology Programme, The Babraham Institute, Babraham Research Campus, Cambridge, CB22 3AT United Kingdom.

*,# equal contribution

Correspondence to al989@cam.ac.uk or stephanie.humbletbaron@kuleuven.be

Supplementary Table 1: Disease activity score

Tissue	Score	Criteria
Spleen	1	+200-300% of WT spleen/body weight ratio
	2	+300-400% of WT spleen/body weight ratio
	3	+400-500% of WT spleen/body weight ratio
Weight	1	Weight gain -10-20% bodyweight of wildtype littermates
	2	Weight gain -20-30% bodyweight of wildtype littermates
	3	Weight gain -30-40% bodyweight of wildtype littermates
Skin	1	Light axillary/inguinal erythema, eczema
	2	Light abdominal erythema, eczema
	3	Abdominal erythema, thickened skin, hair loss
Eyes	1	Hair loss around the eyes or eye watery
	2	Squinted eyes or both eyes closed
	3	Encrusted eyelids
Tail	1	Some scaling on tail
	2	Overall scaly tail
	3	Scaly tail with lesions
Ears	1	Ears folded back to head
	2	Dermal thickening
	3	Encrusted ears
Gait	1	Intermittent hunching at rest
	2	Hunching without activity impairment
	3	Prolonged hunching with activity impairment

Supplementary Table 2: Histological classification

Grade	Histological examination
Grade 0	**No significant lesions**, or normal morphology with no significant tissue effects
Grade 1	**Minimal:** Corresponds to a microscopic change that may be barely noticeable or changes so minor, small, or infrequent as to warrant no more than the least assignable grade. This grade was used where ≤10% of the tissue type in the defined area was involved
Grade 2	**Mild:** Corresponds to a microscopic change that was a noticeable but not a prominent feature of the tissue. For focal, multifocal, or diffusely distributed tissue effects, this grade was used where 11-20% of the tissue type in the defined area was involved
Grade 3	**Moderate:** Corresponds to a microscopic change that was a prominent feature of the tissue. For focal, multifocal, or diffusely distributed tissue effects, this grade was used where 21-40% of the tissue type in the defined area was involved
Grade 4	**Marked:** Corresponds to a microscopic change that was an overwhelming feature of the tissue. For focal, multifocal, or diffusely distributed tissue effects, this grade was used where 41-100% of the tissue type in the defined area was involved

Fig. S1. Generation of the Foxp3^{KO} scurfy mice. A) Design of the *Foxp3* locus before and after intercrossing with Flp-deleter mice. **B)** Breeding of *Foxp3$^{KO/KO}$ RAG$^{KO/KO}$* female mice with *Foxp3$^{WT/Y}$ RAG$^{WT/WT}$* male mice to generate male *Foxp3$^{KO/Y}$ RAG$^{KO/WT}$* mice. **C)** Percentage of Foxp3 Treg in total CD4^{+} T cells from spleen and liver in WT mice versus *Foxp3^{KO}* mice (untreated and CTLA4 treated) (left). Flow plots of Foxp3 expression in total CD4^{+} T cells from spleen in WT mice and *Foxp3^{KO}* mice (right).

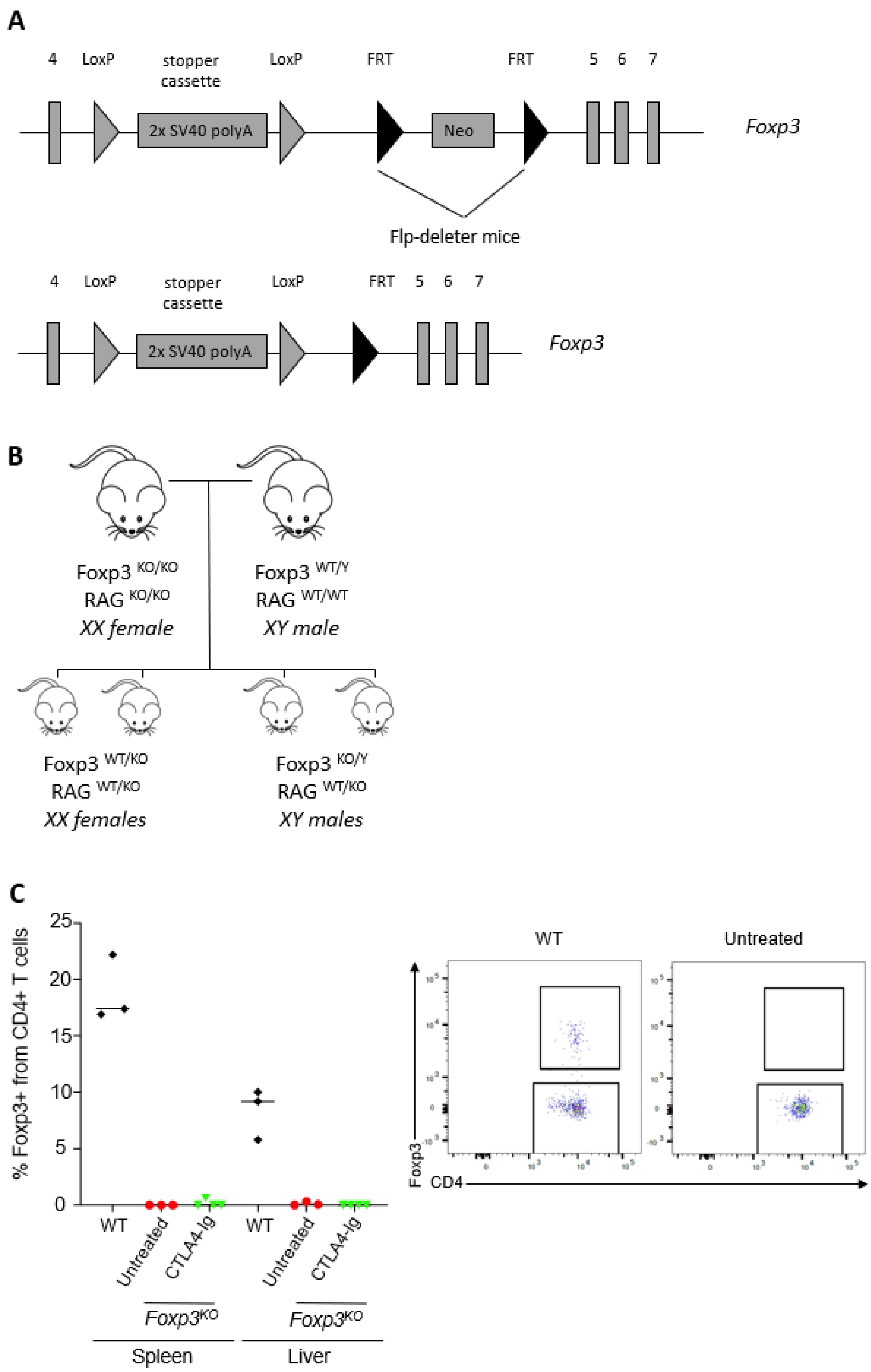

Fig. S2. Representative flow cytometry gating strategies. A) T cell gating strategy. Representative gating strategy performed on wildtype spleen at day 26. **B)** Tbet on CD8^{+} T cell gating strategy. Representative gating strategy performed on wildtype spleen at day 26. **C)** IFNγ and IL4 CD4^{+} T cell gating strategy. Representative gating strategy performed on wildtype spleen at day 26.

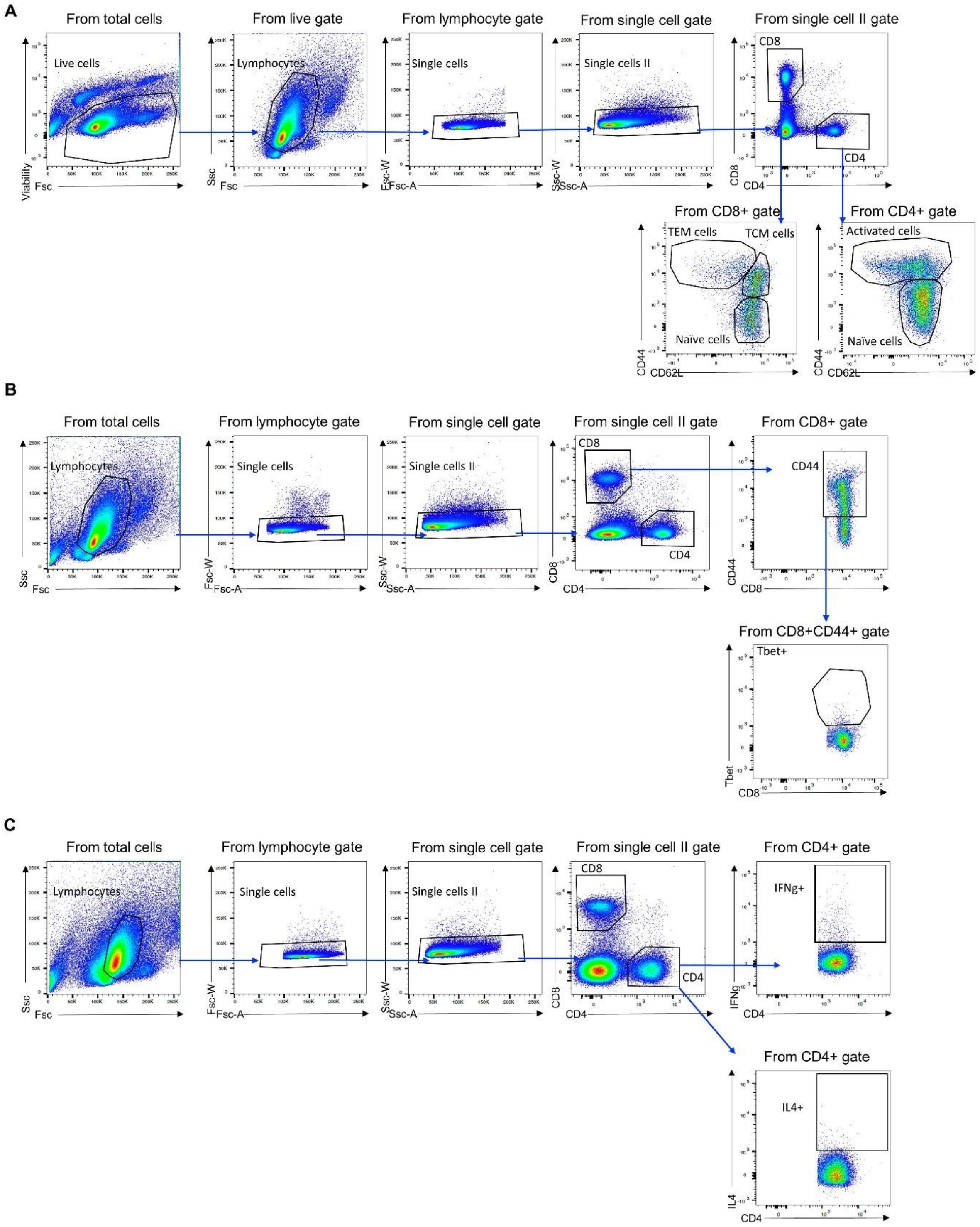

Fig. S3. Experimental design. A) *Foxp3*^{KO} mice and wild-type littermate controls (WT) were followed up either without therapeutic intervention (WT and *Foxp3*^{KO} mice) or under treatment with Rapamycin, CTLA4-Ig, or anti-CD4 antibody. Mice were treated intra peritoneally according to the illustrated schedule, and followed-up with longitudinal assessment of clinical conditions. **B)** The concentration of Rapamycin 24 and 48h after injection was assessed in blood (μg/L).

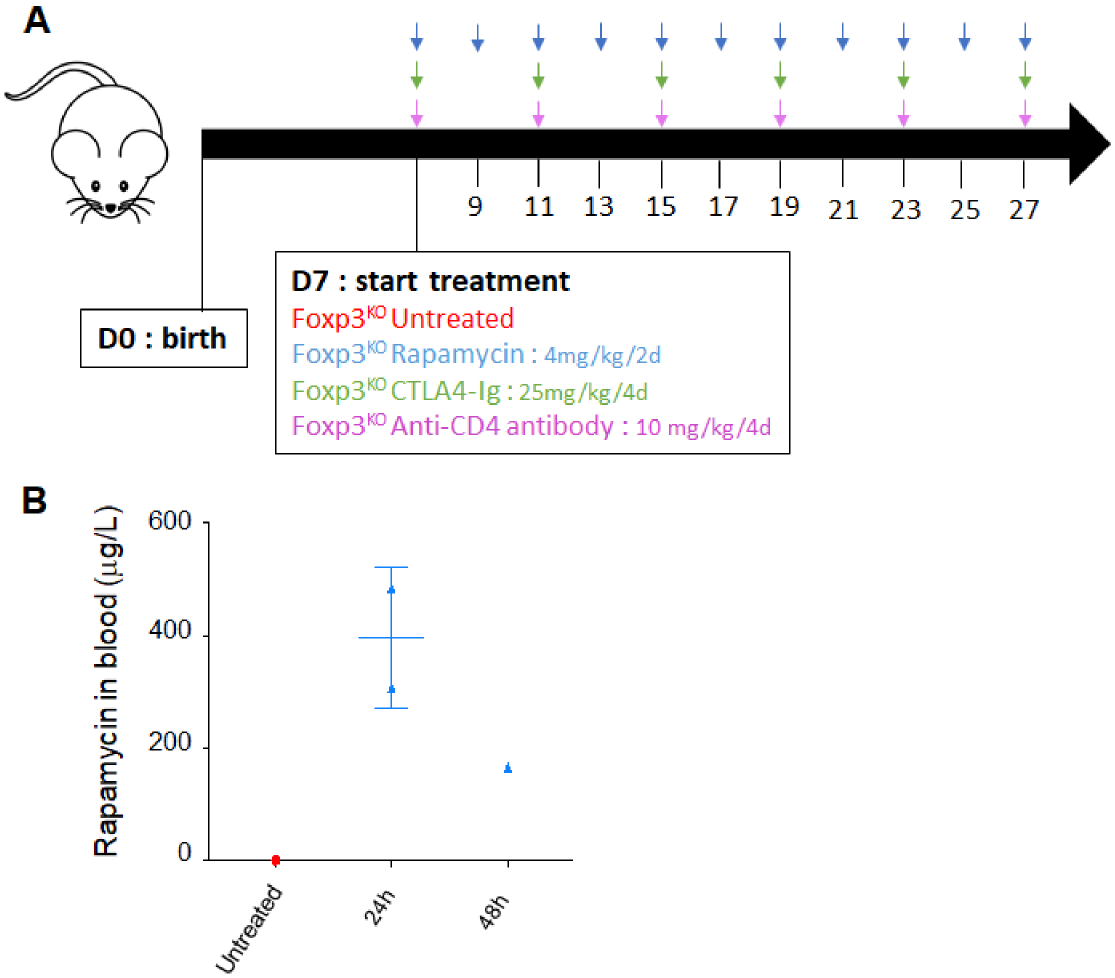

Fig. S4. Representative Flow plots. CD4 and CD8 subsets. Spleen from wildtype mice, *Foxp3*^{KO} untreated, *Foxp3*^{KO} CTLA4-Ig, *Foxp3*^{KO} rapamycin and *Foxp3*^{KO} anti-CD4 antibody were stained at day 26. **A)** CD4 and CD8 from total lymphocyte gate. **B)** Activated cells (CD44^{+}) and naïve cells (CD44^{-}CD62L^{+}) from CD4^{+} T cells gate. **C)** Effector Memory T cells (TEM) (CD44^{+}CD62L^{-}), Central Memory T cells (TCM) (CD44^{+}CD62L^{+}) and naïve cells (CD44^{-}CD62L^{+}) from CD8+ T cells gate. **D)** Tbet expression on CD8^{+} T cell gated on activated CD8^{+} T cells (CD44^{+} CD8^{+} cells). **E)** IFNγ expression on CD4^{+} T cell gated on total CD4^{+} T cells. **F)** IL4 expression on CD4^{+} T cell gated on total CD4^{+} T cells. Representative plot for each condition.

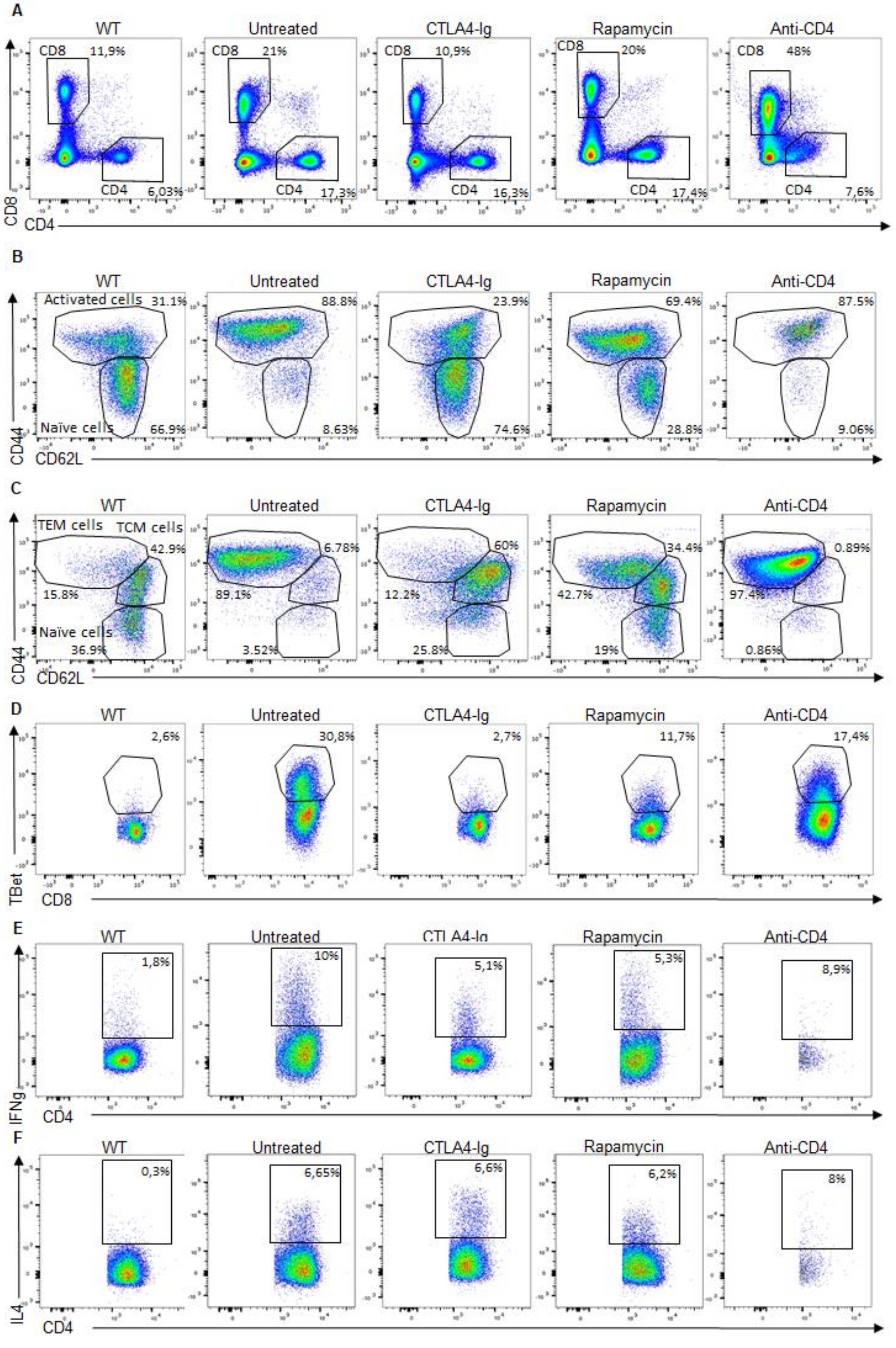
A
WT
Untreated
CTLA4-Ig
Rapamycin
Anti-CD4
CD8 11,9%
CD4 6,03%
CD8 21%
CD4 17,3%
CD8 10,9%
CD4 16,3%
CD8 20%
CD4 17,4%
CD8 48%
CD4 7,6%
CD8
CD4
B
Activated cells 31.1%
Naïve cells 66.9%
88.8%
8.63%
23.9%
74.6%
69.4%
28.8%
87.5%
9.06%
CD44
CD62L
C
TEM cells
TCM cells
42.9%
15.8%
Naïve cells 36.9%
6.78%
89.1%
3.52%
60%
12.2%
25.8%
34.4%
42.7%
19%
0.89%
97.4%
0.86%
CD44
CD62L
D
2,6%
30,8%
2,7%
11,7%
17,4%
TBet
CD8
E
1,8%
10%
5,1%
5,3%
8,9%
IFNg
CD4
F
0,3%
6,65%
6,6%
6,2%
8%
IL4
CD4

Fig. S5. Diverging immunological outcomes of different treatments in Foxp3-deficient mice. *Foxp3*^{KO} mice, untreated or treated with rapamycin, CTLA4-Ig or anti-CD4 antibody, as well as untreated wild-type littermate controls (WT), were assessed on day 26 (WT n=8; *Foxp3*^{KO} untreated n=8, *Foxp3*^{KO} rapamycin n=6, *Foxp3*^{KO} CTLA4-Ig n=9, or *Foxp3*^{KO} anti-CD4 antibody n=5). Flow cytometry results from the lymph nodes for: **A)** The percentage of $CD4^+$ T cells, or **B)** $CD8^+$ T cells from total lymphocytes. **C)** Percentage of naïve ($CD44^-$) and **D)** effector ($CD44^+$) T cells from $CD4^+$ lymphocytes. **E)** Percentage of naïve ($CD44^-$ $CD62L^+$), **F)** central memory (TCM) ($CD44^+CD62L^+$), **G)** TEM ($CD44^+CD62L^-$) and **H)** $Tbet^+$ cells from $CD8^+$ lymphocytes. **I)** Percentage of $CD4^+$ T cells in the lymph nodes expressing IFNγ or **J)** IL4. Violin plot with individual values.

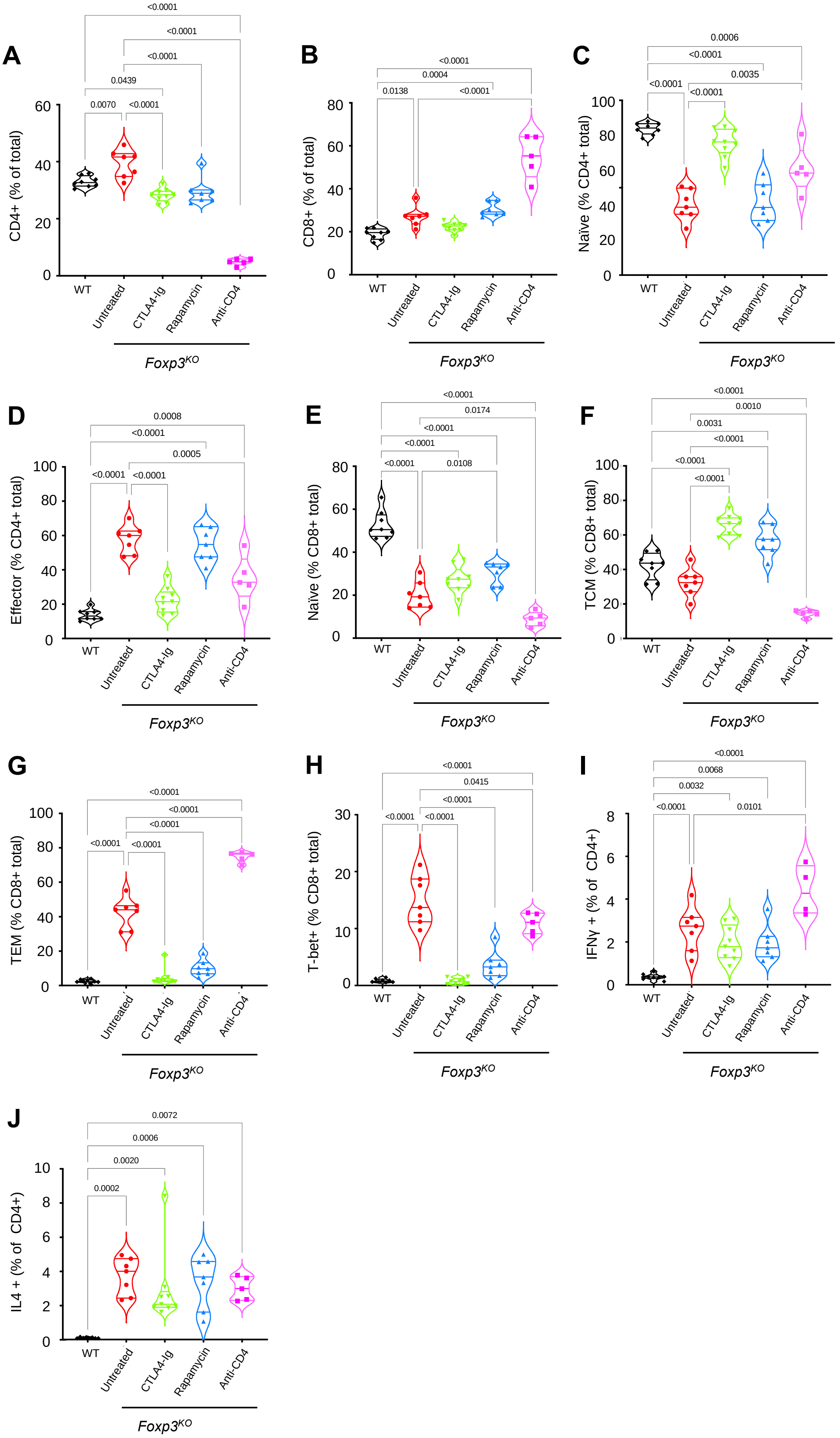
A
CD4+ (% of total)
<0.0001
<0.0001
<0.0001
0.0439
0.0070
<0.0001
B
CD8+ (% of total)
<0.0001
0.0004
0.0138
<0.0001
C
Naïve (% CD4+ total)
0.0006
<0.0001
0.0035
<0.0001
<0.0001
D
Effector (% CD4+ total)
0.0008
<0.0001
0.0005
<0.0001
<0.0001
E
Naïve (% CD8+ total)
<0.0001
0.0174
<0.0001
<0.0001
<0.0001
0.0108
F
TCM (% CD8+ total)
<0.0001
0.0010
0.0031
<0.0001
<0.0001
<0.0001
G
TEM (% CD8+ total)
<0.0001
<0.0001
<0.0001
<0.0001
<0.0001
H
T-bet+ (% CD8+ total)
<0.0001
0.0415
<0.0001
<0.0001
<0.0001
I
IFNγ + (% of CD4+)
<0.0001
0.0068
0.0032
<0.0001
0.0101
J
IL4 + (% of CD4+)
0.0072
0.0006
0.0020
0.0002
WT
Untreated
CTLA4-Ig
Rapamycin
Anti-CD4
Foxp3KO

Fig. S6. Impact of CTLA4-Ig treatment on CD80 and CTLA4 expression in Foxp3-deficient mice. *Foxp3^{KO}* mice, untreated or treated with CTLA4-Ig, as well as untreated wild-type littermate controls (WT), were assessed on day 26 (WT n=3; *Foxp3^{KO}* untreated n=3, *Foxp3^{KO}* CTLA4-Ig n=4). Flow cytometry results from the spleen and lymph nodes for: **A)** The percentage of CD80^{+} cells on inflammatory CD11b^{+} myeloid cells, with representative flow cytometry plots, and **B)** The percentage of CTLA4^{+} cells on conventional CD4+ T cells, with representative flow cytometry plots.

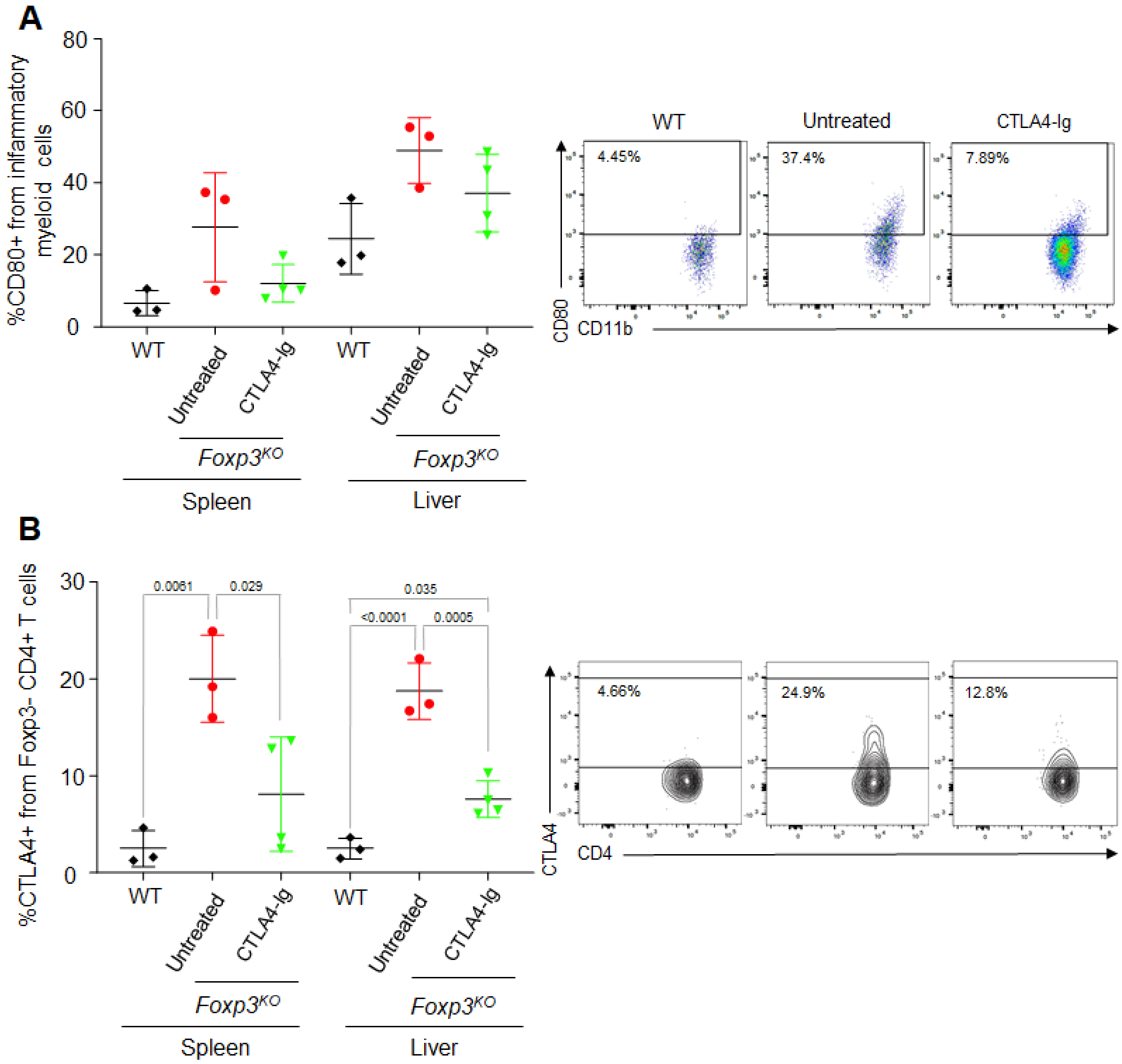

Supplement: Supplementary file 1 — Supplementary file1 (PDF 1091 KB) [file 10875_2023_1462_MOESM1_ESM.pdf]
